# Effect of Nutritional Factors and Copper on the Regulation of Laccase Enzyme Production in *Pleurotus ostreatus*

**DOI:** 10.3390/jof8010007

**Published:** 2021-12-22

**Authors:** Dinary Durán-Sequeda, Daniela Suspes, Estibenson Maestre, Manuel Alfaro, Gumer Perez, Lucía Ramírez, Antonio G. Pisabarro, Rocío Sierra

**Affiliations:** 1 Product and Process Design Group, Department of Chemical and Food Engineering, Universidad de los Andes, 111711 Bogotá, Colombia; d.suspes2053@uniandes.edu.co (D.S.); stibensonmac@gmail.com (E.M.); rsierra@uniandes.edu.co (R.S.); 2 Institute for Multidisciplinary Research in Applied Biology, Public University of Navarre, 31006 Pamplona, Spain; manuel.alfaro@unavarra.es (M.A.); gumer.perez@unavarra.es (G.P.); lramirez@unavarra.es (L.R.)

**Keywords:** multi-copper oxidase, copper-transporter, nutrient-sufficient conditions, white-rot fungus

## Abstract

This research aimed to establish the relationship between carbon–nitrogen nutritional factors and copper sulfate on laccase activity (LA) by *Pleurotus ostreatus*. Culture media composition was tested to choose the nitrogen source. Yeast extract (YE) was selected as a better nitrogen source than ammonium sulfate. Then, the effect of glucose and YE concentrations on biomass production and LA as response variables was evaluated using central composite experimental designs with and without copper. The results showed that the best culture medium composition was glucose 45 gL^−1^ and YE 15 gL^−1^, simultaneously optimizing these two response variables. The fungal transcriptome was obtained in this medium with or without copper, and the differentially expressed genes were found. The main upregulated transcripts included three laccase genes (*lacc2*, *lacc6*, and *lacc10*) regulated by copper, whereas the principal downregulated transcripts included a copper transporter (*ctr1*) and a regulator of nitrogen metabolism (*nmr1*). These results suggest that Ctr1, which facilitates the entry of copper into the cell, is regulated by nutrient-sufficiency conditions. Once inside, copper induces transcription of laccase genes. This finding could explain why a 10–20-fold increase in LA occurs with copper compared to cultures without copper when using the optimal concentration of YE as nitrogen sources.

## 1. Introduction

Fungal laccases are glycosylated, multi-copper oxidases that catalyze the oxidation of hydroxyl functional groups on various substrates and the molecular oxygen reduction to water [1,2,3]. As laccases can oxidize phenolic and non-phenolic compounds, they are attractive in many biotechnological processes such as bioremediation, wastewater treatment, nanobiotechnology, biofuel production, pharmaceuticals, and the food industry [4,5].

*Pleurotus ostreatus* is a white-rot fungus biotechnological model for studying and producing fungal laccases [6]. This fungus is easily cultivable on several synthetic or natural media [7], its genome has been decoded [8], and it contains a laccase multi-gene family [9]. However, laccase production in *P. ostreatus* cultures is affected by complex and not fully understood gene expression regulatory mechanisms at multiple levels.

Twelve laccase genes have been identified in *P. ostreatus* monokaryotic (mk) strains, mkPC15 and mkPC9 [10] distributed across several chromosomes: seven on chromosome VI (*lacc1*, *lacc4*, *lacc6*, *lacc7*, *lacc9*, *lacc10*, and *lacc11*), two on chromosome XI (*lacc5* and *lacc12*), and one on chromosomes IV, VII, and VIII (*lacc3*, *lacc8*, and *lacc2*, respectively) [11]. Their physiological and functional roles and the consequences of their clustering are still under study.

Laccase gene expression regulation is controlled by *cis*-acting elements promoter upstream of the transcriptional start [12]. In addition to the TATA and CAAT boxes, other *cis*-acting promoter elements differ for each laccase gene on *P. ostreatus*. They can be divided into three groups: (i) response to nutrient-sufficient conditions (carbon and nitrogen sufficient) including catabolic responsive elements (CREs) and nitrogen binding site (NIT); (ii) response to inducers, such as metal responsive (MRE), xenobiotic responsive (XRE), and antioxidant responsive elements; (iii) response to stress, including heat shock sequence (HSE), and stress responsive elements (STREs) [13,14]. The first and second groups could be stimulated by changing the culture medium’s composition; however, these changes are insufficient to explain the transcriptional profile of laccases in different culture conditions [15,16]. In addition, there is a lack of knowledge about how other trans-regulatory factors can interact and intervene at this regulation level.

Other laccase regulation mechanisms occurring at the post-transcriptional and post-translational levels could explain changes in the molecular weight of laccase isoenzymes or the heterogeneity observed in the electrophoretic migration of their isoforms [17]. Most isolated and characterized laccases have an apparent molecular weight ranging from 60 to 85 kDa. Although almost all are monomers, Lacc2 can form complex heterodimers [12,13] with a large subunit formed by at least two alternatively spliced variants of the *lacc2* gene, and small subunits produced by translating two different genes [17,18]. Concerning the post-translational regulation level, N-glycosylation is the main post-translational modification in fungal laccases [19]. N-glycosylation is critical in several biochemical aspects, such as the folding, location, and catalytic activity, of these enzymes [20,21]. On the one hand, N-glycosylation is the main challenge to produce laccases in the heterologous system [22]. On the other hand, these modifications are also affected by the fungal growth conditions and culture medium composition [23].

Copper is a transcription inducer for some laccase genes and a cofactor in their catalytic center. This metal is present in the environment, and it is taken up by high-affinity transmembranal copper transporters of the CTR family [24].

Because of all these regulatory levels, a wide range of maximum laccase activities have been reported for submerged fermentation processes aimed at the industrial production of enzymes or enzymatic cocktails to be used in the treatment of lignocellulose wastes in industrial processes.

*P. ostreatus* senses carbon and nitrogen sources and concentrations through different mechanisms and pathways [25] that differentiate and respond to two nutrient conditions (i.e., sufficient and limited), different for each organism [26]. Catabolic repression mechanisms are activated specifically for these nutrient-sufficient conditions when the carbon or nitrogen environmental concentration is high. For carbon, the mechanism, called carbon catabolite repression (CCR) [27], turns on/off certain enzymes upon the availability or scarcity of this sugar [28]. CCR is regulated primarily by the transcription factor Cre [29]. On the other hand, for nitrogen sources, the mechanism is called nitrogen metabolite repression (NMR), and it guarantees the preferential use of ammonium (NH_4_^+^) or L-glutamine as nitrogen sources [30,31]. In filamentous fungi, this mechanism is controlled by the transcription factor Nmr [32]. Both catabolic processes, CCR and NMR have been described in other fungi, are redundant, and have multiple activations or inhibition checkpoints [33,34,35].

As systematic research on the interactions between nutritional factors on the laccase activity of *P. ostreatus* is currently scarce, this work aimed to consider the interaction between the availability of nutritional factors (i.e., carbon and nitrogen) and copper sulfate at 1 mM to determine how the medium composition affects laccase induction by copper. The results to be presented indicate that carbon and nitrogen availability influence the laccase activity only when copper is present in a nutrient-sufficient condition where the transcription of *nmr1* and copper transporters are upregulated.

## 2. Materials and Methods

### 2.1. Chemicals

Copper (II) sulfate pentahydrate, CuSO_4_∙5H_2_O (>98.0%); ammonium sulfate, (NH_4_)_2_SO_4_ (99%); calcium chloride, CaCl_2_ (99.9%); magnesium sulfate heptahydrate, MgSO_4_∙7H_2_O (99%); potassium chloride, KCl (99%); potassium phosphate monobasic, K_2_HPO_4_ (99%); thiamine hydrochloride (99%) sodium citrate dihydrate HOC(COONa)(CH_2_COONa)_2_∙2H_2_O (>99%), and sodium hydroxide anhydrous pellets, NaOH (>98%) were purchased from Merck (Darmstadt, Germany). The citric acid (99.5%), malt extract agar, and yeast extract were supplied by Scharlau (Barcelona, Spain). 2,2′-Azino-bis(3-ethylbenzthiazoline-6-sulfonate), ABTS (>98.0%), 3,5-dinitrosalicylic acid, and DNS (>98.0%) were manufactured from Sigma–Aldrich, St. Louis, MO, USA. D(+)-Glucose anhydrous PA-ACS and sodium acetate anhydrous (99.9%) were obtained from PanReac AppliChem (Barcelona, Spain). Acrylamide and bis-acrylamide solution (30%, 29:1), 0.5 M Tris-HCl, pH 6.8, 1.5 M Tris-HCl, pH 8.8 solutions were provided for Bio-Rad, Alcobendas, Spain.

### 2.2. Fungal Strain and Culture Conditions

The *Pleurotus ostreatus* strain ANDES-F515, provided by the Laboratory of Mycology and Phytopathology of the Universidad de Los Andes-LAMFU and deposited in the ANDES Natural History Museum (MHN ANDES), was isolated in the Bosque de la Merced, Santa Bárbara village, Bojacá, Cundinamarca, Colombia. The dikaryotic mycelium was maintained and conserved on malt extract agar at 4 °C, with periodic replication of the growth zone for eight days of incubation at 25 °C in the dark.

The submerged fermentation (SmF) cultures were performed in 250 mL flasks containing 100 mL of culture medium. For the experiments made using an inorganic nitrogen source 1.0 or 10.0 gL^−1^ of (NH_4_)_2_SO_4_ were added to a minimum salt medium (MSM) composed of 0.5 gL^−1^ K_2_HPO_4_, 0.25 gL^−1^ MgSO_4_∙7H_2_O, 0.1 gL^−1^ CaCl_2_, 0.5 gL^−1^ KCl, 0.5 gL^−1^ thiamine, 2.1 gL^−1^ citric acid, 2.94 gL^−1^ sodium citrate, and 6.5 final pH adjusted with 0.1 M NaOH. For the experiments made using an organic nitrogen source, yeast extract 5 or 10 gL^−1^, and glucose were the only medium components. Glucose (0.5, 1.0, 10, or 20 gL^−1^) was added to achieve different C:N ratios in both experimental conditions.

The elemental compositional analysis of yeast extract (YE) used in this study was performed and revealed a C:N ratio estimated at 4:1.

Only glucose and YE were used in the culture media to evaluate models of surface response optimization using a central composite experimental design. The composition of the media were pairwise combinations of glucose (23.8, 30.0, 45.0, 60.0, or 66.2 gL^−1^) and YE (4.4, 7.5, 15.0, 22.5, or 25.6 gL^−1^) according to the results from the experimental design shown in Section 3.2. (Table 3). All combinations were tested in the absence or presence of 1 mM CuSO_4_∙5H_2_O (0.25 gL^−1^).

All SmF cultures were inoculated with five 4 mm diameter plugs taken from the growth zone of eight-day malt extract agar plates cultures and incubated at 25 °C, 150 rpm, in the dark for 21 days. All experiments were performed in duplicate.

### 2.3. Biomass Production

Fungal biomass production was gravimetrically determined at 21 days of culture. The culture was filtered through a previously dried and weighed filter paper using a vacuum filter system. The collected, filtered mycelium was dried at 45 °C for 72 h and weighed [36].

### 2.4. Biochemical Analyses

#### 2.4.1. Spectrometric Analyses

Laccase activity was determined with ABTS (2,2-azino-bis(3-ethylbenzthiazoline-6-sulfonate)) as the substrate [37]. The assay mixture contained 1 mM ABTS, 20 mM sodium acetate buffer (pH 5.0), and 10 μL aliquots of an appropriately diluted enzyme sample. Oxidation of ABTS was monitored by following the increase in *A*_436_ (ε 29.3 mM^−1^cm^−1^). A laccase activity unit was defined as the enzyme required to oxidize 1 μmol ABTS per minute at 25 °C. Reducing sugars were measured using the 3,5-dinitrosalicylic acid (DNS) reagent by DNS method at 540 nm [38] using glucose as a reference for the calibration curve. Bradford protein assays (Bio-Rad, Alcobendas, Spain) were used for total protein quantification. According to the manufacturing instructions, standards and samples were mixed with Coomassie Blue Assay Reagent G-250. Each reaction was measured at 595 nm [39].

#### 2.4.2. Zymogram

Non-denaturing and non-reducing electrophoresis conditions (native-PAGE) were used to visualize the isoenzymes present in samples from the liquid phase of the culture medium [40]. The stacking and separating gels contained 4% and 9% (*v*/*v*) acrylamide/bis-acrylamide and were adjusted with 25% (*v*/*v*) of buffer solution at 0.5 M Tris-HCl, pH 6.8, and at 1.5 M Tris-HCl, pH 8.8, respectively. The running buffer was 0.025 M Tris–0.192 M Glycine pH 8.3. Samples were mixed with native sample buffer containing 62.5 mM Tris-HCl, pH 6.8, 40% glycerol, 0.01% bromophenol blue. Native-PAGE was running in the Mini-PROTEAN Tetra Vertical Electrophoresis Cell (Bio-Rad, Alcobendas, Spain) at 100 V. The isozymes were revealed through 30 min gel staining with ABTS 2 mM.

### 2.5. Microscopy Observation

**Figure 1:**
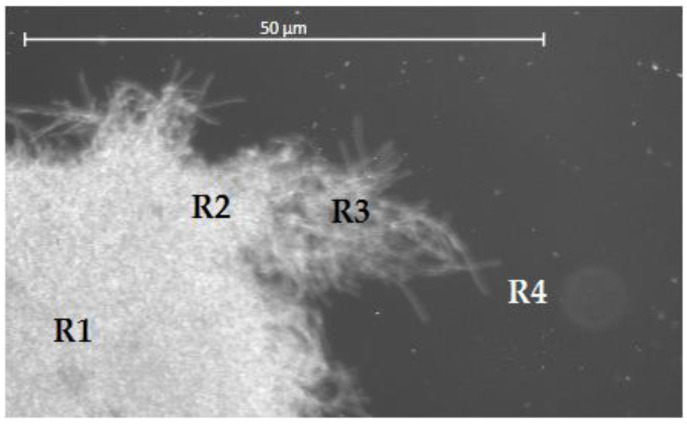
View of half a pellet from *P. ostreatus* in submerged culture. R1 is the central region of the pellet. R2 is the region of the peripheral hyphae. In R3, the most peripheral hyphae are far from R2. In R4, some hyphae in the peripheral region are separated. R4 corresponds to the so-called terminal hyphae in this study.

Samples with tiny pellets produced in the culture media up to day 7 were taken with a plastic Pasteur pipette (graduated from 1 mL). The samples were placed in a 1 mL Eppendorf tube and then carefully took a single pellet (Figure 1) to be placed between a slide and coverslip glass and then placed under a Zeiss Model AxioSkop HBO 50 W Mercury Upright Fluorescence Microscope connected to a ZEISS Axiocam 208 color/202 mono camera and ZEN 2.3 lite software. The first clamp connection distributed in a terminal hypha (Figure 1, R4) of the pellet was just searched from the apical cell to the sub-apical cell. Three clamp connections distributed in three different terminal hyphae were searched for in each pellet. The clamps connections were centered at 100× magnification and individually photographed by phase-contrast microscopy.

### 2.6. RNA Isolation and Transcriptional Analyses

The fungal biomass produced in the cultures containing 45 gL^−1^ glucose and 15 gL^−1^ YE, with or without 1 mM CuSO_4_, were harvested on day 12 of culture for RNA isolation. The mycelium was collected by filtration, immediately frozen in liquid nitrogen, and ground to a fine powder in a mortar. Then, 100 ng of the powder were transferred to a 1.5 mL microcentrifuge tube for total RNA extraction using a Fungal RNA EZNA Kit (Omega Bio-Tek, Norcross, GA, USA) according to the manufacturer guidelines. Finally, the integrity and quantity of RNA were validated using Bioanalyzer (version 2100) and Qubit 2.0 fluorometer.

#### 2.6.1. Real-Time qPCR

Reverse transcription (RT) was performed using 800 ng per sample of the total RNA to obtain cDNA in a 20 µL volume using an iScript cDNA synthesis kit (Bio-Rad, Alcobendas, Spain). The complete reaction mix was incubated according to manufacturing instructions in a thermal cycler (MJ Research, Inc., Saint-Bruno-de-Montarville, QC, Canada). RT products were diluted 1:20 and kept at −20 °C until real-time qPCRs were performed in a CFX96 real-time system (Bio-Rad Laboratories, Hercules, CA, USA) using SYBR green dye to detect the product amplification [15]. Each reaction mixture was set to a final volume of 20 µL containing 10 µL iQ SYBR green Supermix (Bio-Rad Laboratories), 2 µL of 5 µM stock forward and reverse primers (Table 1), 1 µL of RT product diluted, and 5 µL of sterile water. Cycling conditions were as follows: denaturation 5 min at 95 °C, 40 cycles for 15 s at 95 °C, 30 s at 63 °C, 15 s at 72 °C, and a final step using a linear gradient increase of 0.5 °C every 5 s from 65 to 95 °C. Each reaction was performed in triplicate, and non-template controls (NTCs) were included for each primer set. An experimentally validated inter-plate calibrator (IPC) was used to compensate for inter-plate variations. Crossing-point (Cp) values and relative fluorescence units were recorded, and the latter was used to calculate amplification efficiencies by linear regression using the LinReg program [41]. *Sar1*, *gapdh1*, *actin1*, and *pep* were used as reference genes for normalization (Table 1).

#### 2.6.2. mRNA-Seq Analysis

Illumina compatible libraries were prepared to be sequenced using the Illumina Nova Seq 6000 system from an mRNA isolate originating from RNA total. Following sequencing, RNA-seq data were filtered for assurance quality using FastQC and trimmed with BBDuk to remove adapters and low-quality reads (https://jgi.doe.gov/data-and-tools/bbtools/bb-tools-user-guide/bbduk-guide/ (accessed on 21 July 2020)). The sequence available at www.genome.jgi.doe.gov/PleosPC15_2/PleosPC15_2.home.html (accessed on 7 December 2021) was used as a *P. ostreatus* genome reference to align the resulting reads STAR v2.3.16 [42]. The parameters used to achieve a single hit mapping were: --outReadsUnmapped Fastx --outFilterMismatchNoverLmax 0.04 --outFilterMultimapNmax 1. The mkPC15 v2.0 reference genome was assembled entirely in twelve scaffolds (34.3 Mb genome size) [43]. In total, 12,330 genes were annotated in this genome [44]. The expression levels were quantified using the Python script rpkmforgenes.py (www.sandberg.cmb.ki.se/media/data/rnaseq/rpkmforgenes.py (accessed on 30 July 2020)) to calculate values of reads per kilobase of transcript per million mapped reads (RPKM).

### 2.7. Differential Gene Expression Analysis and Gene Annotation

Differentially expressed gene (DEG) analyses were performed using the EdgeR Bioconductor package and a dispersion parameter of 0.1. These analyses determined the transcriptional changes in the two culture conditions by comparing the gene expression values based on transcript reads. The gene expression values with Log_2_ fold read changes with a *p*-value < 0.01 and an FDR (false discovery rate) < 0.05 as the cut-off for statistical significance were used. A DEG with log_2_ Fold Change ≥ −2 was established as an upregulated gene, and log_2_ Fold Change ≤ −2 was established as a downregulated gene.

Gene annotations were based on the Joint Genome Institute (JGI) automated annotation to transcript identifications of the mkPC15 v2.0 reference genome. JGI automated annotation uses the following databases: Gene Ontology (GO), Kyoto Encyclopedia of Genes and Genomes (KEGG), InterPro (IPR), Eukaryotic Orthologous Groups (KOG) and Enzyme Commission numbers (EC number), Transporter Classification Database (TCDB), Carbohydrate-Active EnZymes (CAZymes) and MEROPS database (proteolytic enzymes) [45]. In the case of unannotated genes, the Basic Local Alignment Search Tool (BLAST) was used to find local similarity between JGI sequences unannotated and The National Center for Biotechnology Information (NCBI) standards database sequence [46,47].

### 2.8. Statistical Analyses

Minitab^®^ version 18 software was used to construct the statistical design, evaluate statistical significance, obtain the regression models, and find the simultaneous local optimum of one or more response variables.

#### 2.8.1. Principal Component Analysis (PCA)

A principal component analysis (PCA) was performed to explore the correlations between nutritional variables, glucose, ammonium sulfate, and yeast extract with the production of fungal biomass and laccase activity with and without copper sulfate. It was conducted using the data shown in Section 3.1 (Table 2).

#### 2.8.2. Central Composite Design (CCD)

The central composite experimental design allows estimating the curvature of a response surface for a chosen response variable (*y*) and the terms (*β y ε*) of Equation (1) or Equation (2) regression model that allows for the calculation of an optimal point.

(1) $$y=\beta_{0}+\beta_{1}x_{1}+\;\beta_{2}x_{2}+\varepsilon$$

(2) $$y=\beta_{0}+\beta_{1}x_{1}+\;\beta_{2}x_{2}+\beta_{11}x_{11}^{2}+\beta_{22}x_{22}^{2}+\beta_{12}x_{1}x_{2}+\varepsilon$$

Factorial ANOVAs were performed to determine the main effect and interaction effects on chosen variables on the response variable laccase activity and biomass production.

## 3. Results

### 3.1. Effects of Inorganic and Organic Nitrogen Source on Laccase Activity

The effect of nitrogen source and carbon–nitrogen ratio (C:N) were evaluated in submerged fermentation (SmF) cultures of *P. ostreatus*. The composition of these culture media, fungal biomass production, final glucose concentration, and maximum laccase activity at 21 days of fungal growth are shown in Table 2. Higher biomass production and laccase activity were obtained in cultures with YE as the nitrogen source, whereas the highest final glucose concentration, lower biomass, and laccase activities lower than 10 UL^−1^ were obtained when the nitrogen source was ammonium sulfate. When the nitrogen source was YE, the maximum laccase activity was 16-fold higher on average than the observed in similar cultures with ammonium sulfate as a nitrogen source. The highest laccase activity was detected in the cultures with the highest glucose concentration (20 gL^−1^) and 14:1 C:N in their composition.

**Figure 2:**
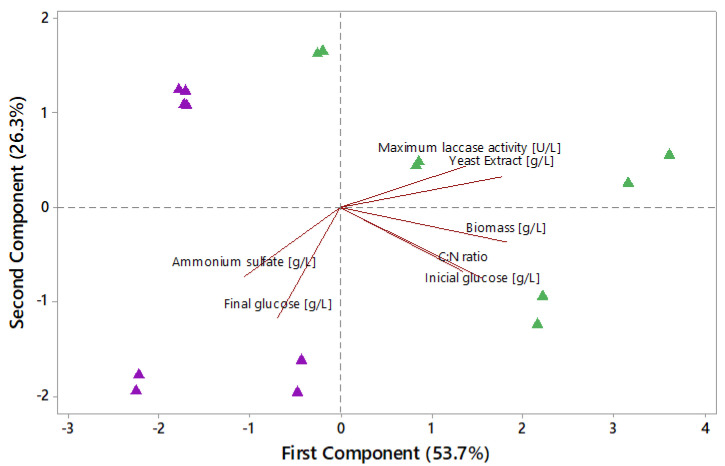
Principal component analysis (PCA) of biological parameters of growth of *P. ostreatus* in culture media with ammonium sulfate or yeast extract in SmF. The purple triangles represent culture media with ammonium sulfate and the green triangles media with yeast extract.

The principal components analysis (PCA) results are shown in Figure 2. Most data were grouped into three principal components that explained 93.3% of the variation in the data with the first two dimensions (PC1 and PC2) accounting for most of the correlation of all measured variables (53.7% and 26.3%, respectively). The maximum laccase activity correlated with the highest values of YE in the culture medium and high initial glucose concentrations positively correlated with C:N ratios and fungal biomass production (first component). The ammonium sulfate correlates with the highest undigested glucose left in the culture media (second component).

**Figure 3:**
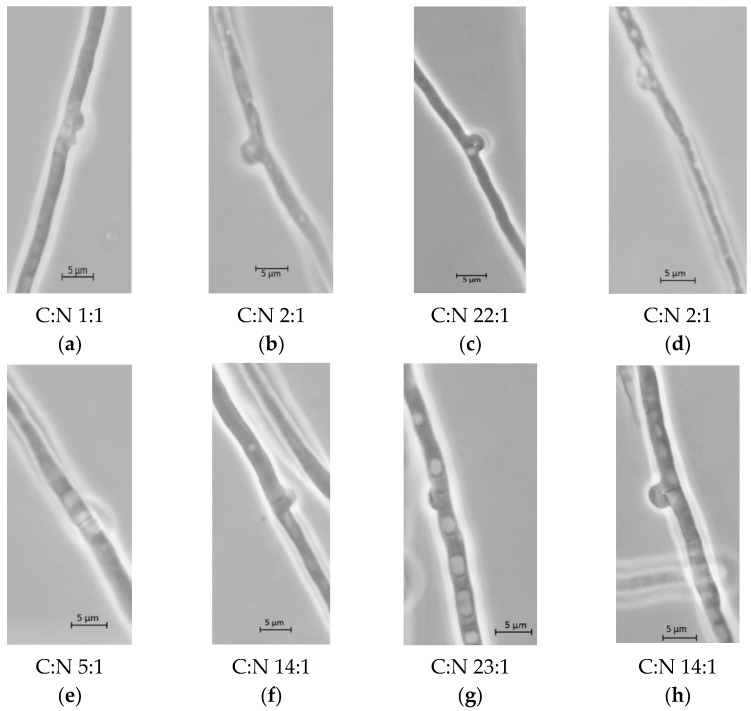
Clamp connection in terminal hyphae from *P. ostreatus* cultures at seven days of culture using different media compositions: (**a**) glucose and ammonium sulfate 0.5 and 1 gL^−1^; (**b**) glucose and ammonium sulfate 1 and 1 gL^−1^; (**c**) glucose and ammonium sulfate 10 and 1 gL^−1^; (**d**) Glucose and ammonium sulfate 10 and 10 gL^−1^; (**e**) glucose and YE 5 and 1 gL^−1^; (**f**) glucose and YE 5 and 10 gL^−1^; (**g**) glucose and YE 5 and 20 gL^−1^; (**h**) glucose and YE 10 and 20 gL^−1^.

To explore if nutrient-sufficient or nutrient-limited culture conditions induced morphological changes in the mycelium, we studied the distribution of clamp connections in apical hyphae of the fungal pellets. Figure 3 shows some representative pictures of these apical hyphae. Clamp connections in different developmental states were found in cultures containing ammonium sulfate (Figure 3a–d) or YE (Figure 3e–h). Differences in the hyphal shape and frequency of vacuoles were found in both apical and sub-apical cells. When YE was used, the highest nitrogen-limited condition (Figure 3g) produced fungal hyphae with the highest vacuolated pattern. This nutrient-limited condition showed the lowest laccase activity among YE cultures (Table 2).

Although vacuoles were observed in the hyphae when ammonium sulfate was used, the vacuolated patterns were variable. Fewer vacuoles occurred in the medium with the highest glucose concentration and the higher ratio C:N (Figure 3c) than those observed in a medium with the same glucose concentration and a lower C:N ratio (Figure 3d). However, laccase activity (Table 3) in both conditions was similar. Finally, when the vacuolation patterns of fungal hyphae from two YE nutrient-sufficient conditions were compared, both nitrogen and glucose were needed to increase laccase activity by *P. ostreatus*. Few vacuolated hyphae were observed in the cultures with nitrogen-sufficient conditions (Figure 3e). This condition showed the highest laccase activity per gram of biomass in the culture, while some vacuolated patterns were observed in fungal hyphae with the highest laccase activity grown in carbon-sufficient conditions (Figure 3g).

### 3.2. Effect of Glucose and Yeast Extract Concentrations on Laccase Activity

Different YE and glucose concentrations were tested to determine the culture composition that maximizes biomass and laccase production in SmF cultures without or with 1 Mm copper sulfate as a laccase inducer. The central composite experimental design (CCD) results are shown in Table 3 and Figure 3.

The CCD model fit to a quadratic model explained (*p*-value < 0.05) the changes in biomass production as a function of glucose and YE concentrations with *R*^2^ 0.8231 and 0.8035 for biomass production with and without copper sulfate, respectively. On the other hand, the laccase activity induced by copper sulfate was explained by a quadratic model (*p*-value < 0.05) with glucose and YE concentration as varying factors (*R*^2^ 0.9560); however, in the absence of a laccase inducer (i.e., the copper salt) in the culture, the media composition was statistically insignificant (*p*-value > 0.05) and insufficient (*R*^2^ 0.4039) to explain this response variable.

**Figure 4:**
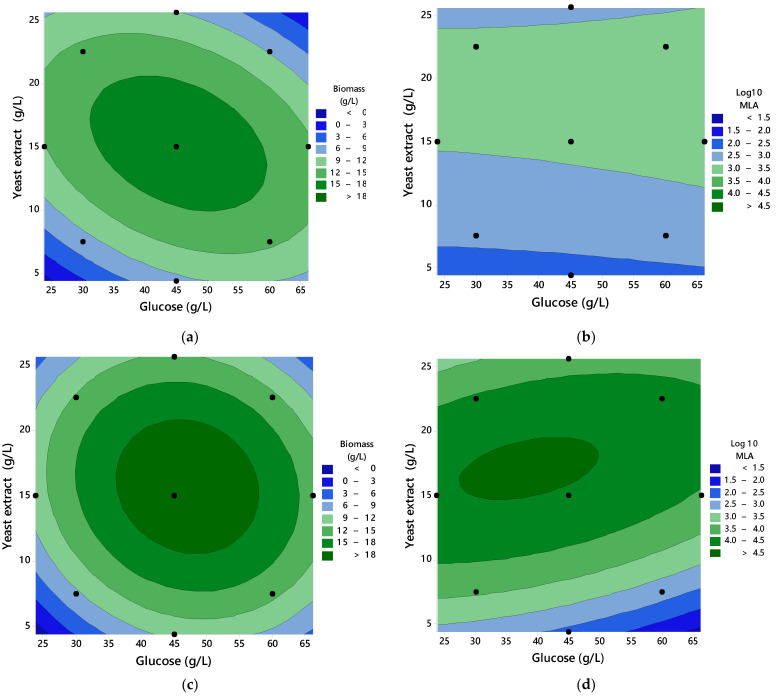
Contour plots of YE and glucose concentration vs. biomass and maximum laccase activity: (**a**) Biomass production in cultures without copper sulfate; (**b**) maximum laccase activity (MLA) as Log_10_ MLA (UL^−1^) production in cultures without copper sulfate; (**c**) biomass production in cultures with copper sulfate; (**d**) maximum laccase activity (MLA) as Log_10_ MLA (UL^−1^) production in cultures with copper sulfate.

According to the CCD models, the contour plots (Figure 4a,c) showed that biomass production was affected by the factors square (glucose and YE concentrations), both in the absence or presence of copper sulfate. The optimum point for biomass production contained 45 and 15 gL^−1^ glucose and YE, respectively (experiment noted as GY4515) for cultures with and without copper. Optimum biomass production in the culture medium with copper was experimentally slightly better, and the model successfully predicted this result.

In the case of maximum laccase activity (Figure 4b,d), the CCD model showed that this response variable was affected by the main effect of YE concentration and the interaction between the glucose and YE concentrations. The experimental maximum laccase activity from the culture media GY4515 was close to where the model predicted the maximum laccase activity. The laccase activities from GY4515 with copper sulfate were 1.4–1.5 logarithmic units greater than GY4515 without copper sulfate (Figure 4b,d).

### 3.3. Characterization of the Growth and Laccase Activity of P. ostreatus under Nutrient Sufficient Conditions with and without Copper Sulfate

**Figure 5:**
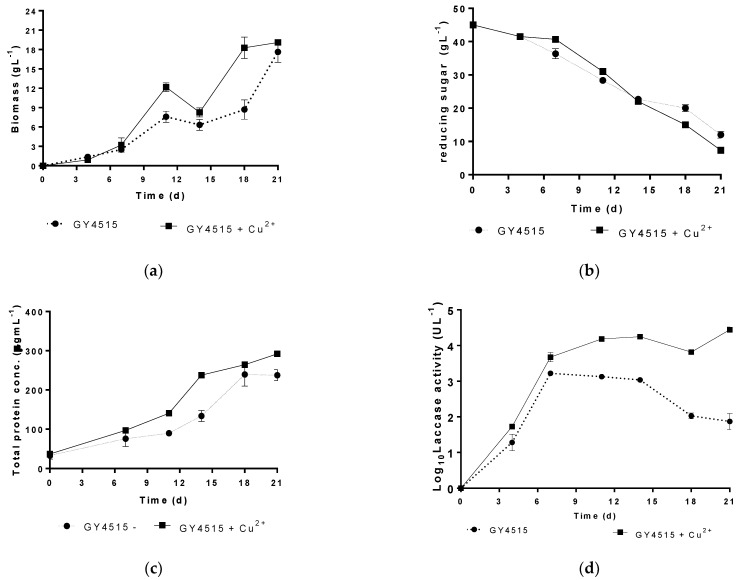
Growth profile of *P. ostreatus* in GY4515 culture media: (**a**) biomass production; (**b**) glucose consumption; (**c**) total protein in culture extract; (**d**) laccase activity (as Log_10_). Black circle—discontinuous line (no copper sulfate); black square—continuous line (copper sulfate 1 mM).

The GY4515 composition medium was selected to characterize the growth of *P. ostreatus* in the presence and absence of 1 mM copper sulfate. For this characterization, biomass production, glucose consumption, total protein, and laccase activity were measured at different culture times until day 21 (Figure 5). The fungal growth profiles in both cultures (Figure 5a) showed two phases characteristic of diauxic growth. The maximum specific growth rates (*µ_max_*) were 0.52 and 0.69 d^−1^ for the copper-free and copper sulfate-containing media. Although *µ_max_* was 32% higher in the presence of copper, the total biomass produced at the end of the culture time (21 d) was similar in both systems (17 and 20 gL^−1^, respectively) (Figure 5a).

The glucose consumption profile was similar in both systems. Glucose concentration decreased with the culture time to a final glucose concentration equivalent to 20% of the initial concentration (Figure 5b). At the same time, the total protein concentration increased in both systems. In the medium with copper sulfate, the total protein was 20% higher than in the culture media without copper (Figure 5c).

The most significant difference between both cultures was the laccase activity profile. While the highest laccase activity values in the medium without copper sulfate were detected before day 12, the highest activity values occurred beyond this day in the media with the laccase inducer. At the end of the culture, the laccase activity decreased in the media without copper, while it continued increasing when the inducer was present. A minimum 10-fold higher laccase activity was obtained by adding 1 mM copper sulfate to the culture media.

### 3.4. Transcriptome Analysis of P. ostreatus under Nutrient Sufficient Conditions with and without Copper Sulfate

**Figure 6:**
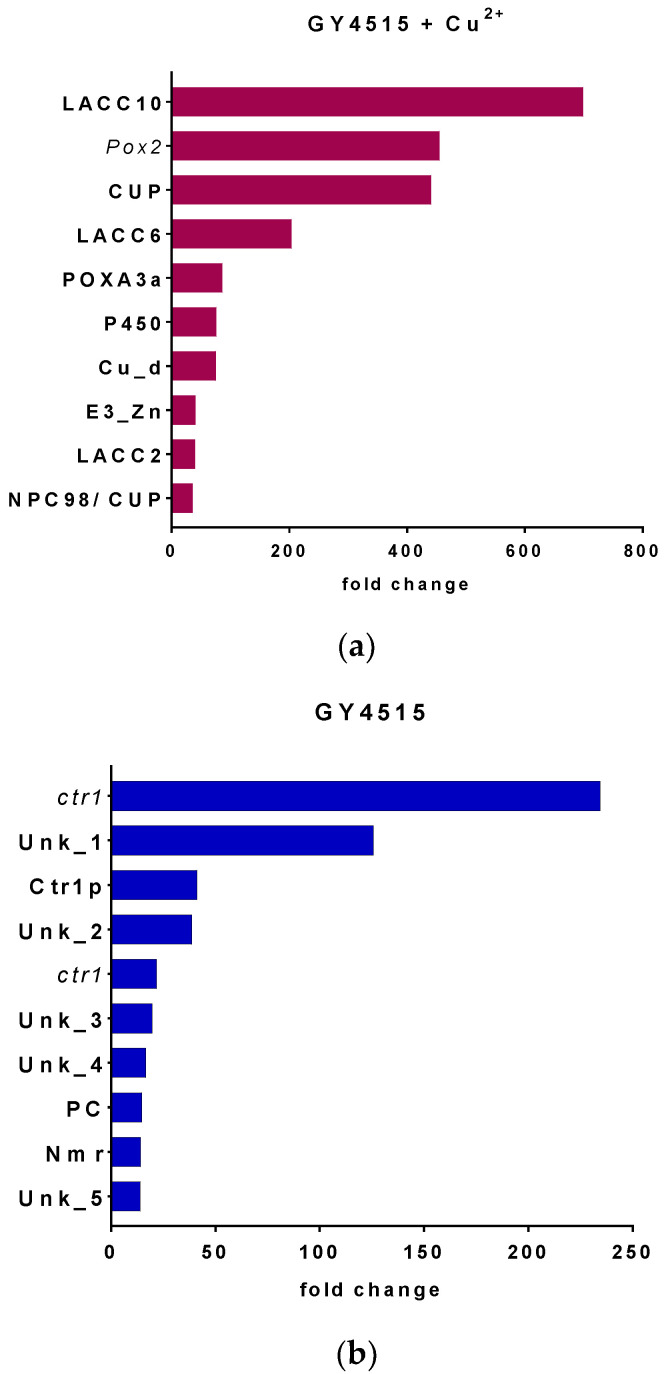
Differential gene expression (DGE) analysis of *P. ostreatus* in culture GY4515 vs. GY4515 + Cu^2+^. The top 10 of upregulated genes (**a**) in the presence and (**b**) in the absence of copper.

The top 10 more differentially expressed genes (up- and downregulated) revealed by the analysis of the *P. ostreatus* transcriptome in the GY4515 culture medium with o without copper sulfate are shown in Figure 6 and Table 4. The laccase genes *lacc10*, *lacc6*, and *lacc2* appeared among the top 10 overexpressed transcripts in the cultures supplemented with copper. Additionally, the transcripts of the small subunit of laccase POXA3a, two cupredoxin, a gene similar to the *pox2* gene, and a transcript coding for a putative copper-binding protein were also among the more overexpressed in these conditions. Conversely, in the absence of copper sulfate, the transcripts of three suspected copper transporters, a lipid transporter, the regulator of nitrogen metabolism Nmr, and five genes with unknown function were among the 10 most overexpressed.

### 3.5. Analysis of Laccase Gene Transcripts

**Figure 7:**
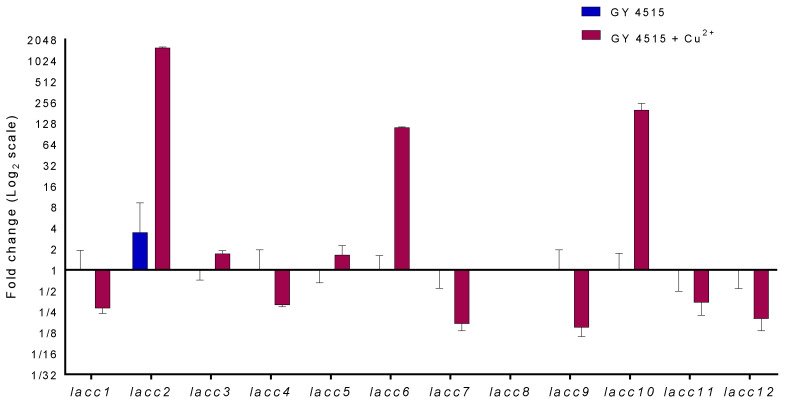
Relative quantification to the 12 *P. ostreatus* laccase genes in GY4515 medium on day 12 of culture. Blue bar: GY4515 without copper sulfate; red bar: GY4515 with copper sulfate, 1 mM.

The relative quantification of the transcripts of the 12 laccase genes annotated in the *P. ostreatus* genome was carried out to determine the genes responsible for the activity in the GY4515 medium with and without copper sulfate. In Figure 7, the results show that in the absence of copper sulfate, the *lacc2* gene transcript was the only one significantly detected, while in the presence of this laccase inducer, the *lacc2*, *lacc6*, and *lacc10* gene transcripts were the most highly upregulated. To a lesser extent, the transcripts of *lacc3* and *lacc5* genes were also overexpressed in the presence of copper sulfate. The *lacc8* gene could not be amplified.

### 3.6. Effect Limited-Nutrition Conditions on Laccase Production

**Figure 8:**
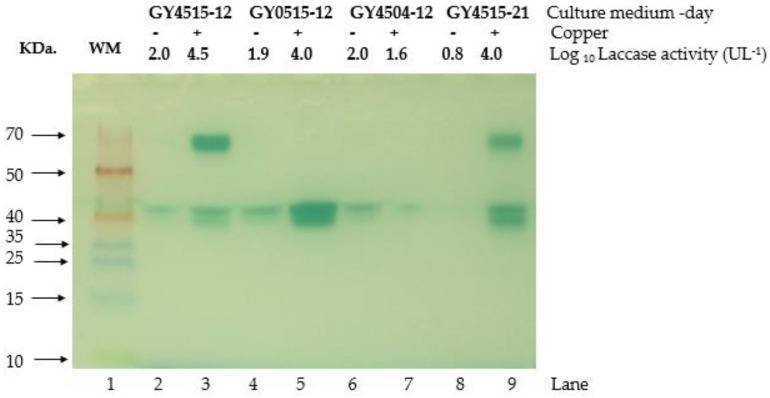
Zymogram of laccases in the supernatant in culture media with different nutrient conditions at 12 or 21 days of culture. Lane 1. Molecular weight marker (WM). Left to right: Lane 2–3 nutrient-sufficient condition GY4515 day 12 (GY4515); Lane 4–5 carbon-limited condition GY0515 day 12 (GY515); Lane 6–7 nitrogen-limited condition GY4504 day 12 (GY4504); Lane 8–9 nutrient-sufficient condition GY4515 day 21 (GY4515-21). Medium without copper (−), with copper (+). Laccase activity is presented as log_10_ (UL^−1^).

As copper-induced laccase activity depended on both glucose and YE concentration, the effects of copper in nutrient-limited conditions were tested. For this, two cultures in nutrient-limited conditions, carbon- and nitrogen-limited conditions, were chosen: the carbon-limited medium was 5.0 gL^−1^ glucose, and 15 gL^−1^ YE (GY0515), whereas the nitrogen-limited medium was 45 gL^−1^ glucose and YE, 4.0 gL^−1^ (GY4504). Figure 8 shows the electrophoretic migration of the laccase activity recovered from the supernatant of the GY4515, GY0515, and GY4504 media at two culture times, 12 and 21 days, in the absence and presence of copper.

In the zymogram (Figure 8), the migration patterns and intensity of the bands changed depending on the presence or absence of copper sulfate, the composition of the culture medium, and the culture day. In the absence of copper sulfate, only two weak bands sized around 40 kDa were present, which coincide with the laccase activity for all these extracts cultures on day 12th of culture around two logarithmic units. The addition of copper sulfate to the nutrient-sufficient medium, GY4515, revealed four bands, two at 70 kDa and the other at 40 kDa (lanes 3 and 9). In these cultures, the laccase activities recovered on days 12th and 21st were more than four logarithmic UL^−1^ (Figure 8, lane 3 and 9). Under carbon-limitation (GY0515), only two strong bands appeared at 40 kDa. In this condition, the laccase activity was around two logarithmic units higher in the copper-containing than in the copper-lacking medium. In contrast, in the nitrogen-limited cultures GY4504, the addition of copper sulfate was insufficient to increase laccase activity. For these cultures, only one weak band appeared at 40 kDa.

### 3.7. Hyphal Morphology of P. ostreatus under Different Nutrient Conditions with Copper

**Figure 9:**
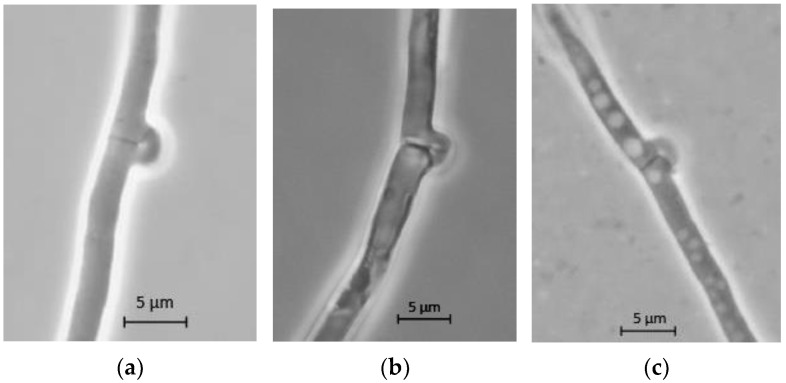
Clamp connections in terminal hyphae of *P. ostreatus* in culture media with different nutrient conditions and copper sulfate 1 mM at the 7th culture day: (**a**) Carbon and nitrogen sufficient condition, GY4515 + Cu^2+^; (**b**) Carbon limited and nitrogen sufficient condition, GY0515 + Cu^2+^; (**c**) Carbon-sufficient and nitrogen-limited condition GY4504 + Cu^2+^.

The hyphal morphology was observed in three selected nutrient conditions with copper: nutrient-sufficient (GY4515), carbon-limited (GY0515), and nitrogen-limited (GY4504). The micrographs of the clamp connections in terminal fungal hyphae at seven days of culture are shown in Figure 9. The results showed that fungal hyphae from all nutrient conditions medium had clamp connections as expected for a dikaryotic strain. Fungal hyphae in nutrient-sufficient condition, GY4515, were no-vacuolated, whereas some level of vacuolation was observed in both nutrient-limited conditions. In hyphae from the carbon-limited medium, GY0515, this level vacuolated pattern was barely observed, while it was clear in hyphae from the nitrogen-limited medium, GY4504

## 4. Discussion

Systematic research on the interactions between nutritional factors on laccase activity production by *P. ostreatus* cultivated on natural substrates is currently scarce; therefore, this work aimed to consider the interaction between two nutritional factors (i.e., carbon and nitrogen concentration) and copper sulfate at 1 mM to determine how the medium composition affects laccase induction by copper in submerged fermentation. Increased fungal biomass production is usually a sign of sufficient nutrient conditions when carbon and nitrogen sources are assimilable in the culture media in the presence of sufficient concentration of other growth factors. Our study chose two nitrogen sources (i.e., ammonium sulfate and YE) to establish which one best increased fungal biomass production and laccase activity in Smf. This approach allowed comparing the performance of nutrient-sufficient, only carbon-sufficient and nitrogen-sufficient conditions, assessing laccase activity in the culture media in the absence or presence of laccase-inducing copper sulfate.

YE was a more assimilable nitrogen source than ammonium sulfate to stimulate biomass and laccase activity in the evaluated culture media conditions; therefore, in subsequent experimentation, glucose and YE concentrations were varied using a central composite experimental design, which applies in the surface response methodology, to optimize the composition of the culture medium for optimal laccase activity. It was found that glucose and YE concentrations did not sufficiently explain the laccase activity in cultures without a copper inducer; however, when copper sulfate was in the culture media, both glucose and YE concentrations influenced this enzymatic activity causing an increase of around 20-fold. Furthermore, from this analysis, it was possible to infer that the organic nitrogen-sufficient conditions incremented laccase production.

Laccase activity in *P. ostreatus* and other white-rot fungi has been frequently reported to be affected by nitrogen sources [48]. Therefore, we tested the effect of ammonium sulfate and yeast extract on laccase activity. We tested different nitrogen sources and C:N ratios. Because the lower C:N ratio indicates more nitrogen disponibility than higher C:N ratios in the culture, our results suggest that laccase activity is dependent on carbon-sufficient and nitrogen-sufficient conditions. In addition, ammonium sulfate was insufficient to increase laccase activity at different nutrient concentrations (C:N ratio). Using yeast extract as a nitrogen source, the magnitude of laccase activity seemed to depend on glucose concentration and C:N ratio. Although in other studies, the increase in ammonium sulfate concentration increased laccase activity in the culture [49], the positive effect of a lower C:N ratio (nitrogen–sufficient conditions) seemed to depend on the complex carbon source and the culture in solid-state fermentation (SSF) [50]. On the contrary, we found a positive effect of yeast extract on laccase activity. This positive effect was also reported for both SmF [51] and SSF [52].

The profiles of fungal growth, laccase activity, laccase gene family expression, and genome-wide gene transcription were studied using high-resolution techniques, such as real-time qPCR and RNA-seq, in the culture medium composition with sufficient carbon and nitrogen. The time course of laccase production during the culture was different in the presence or absence of copper, suggesting that the regulatory mechanisms of laccase activity production are different in both systems. Comparisons showed that the main change in *P. ostreatus* growth with and without copper was laccase activity by the copper-induction of three laccase genes: *lacc2*, *lacc6*, and *lacc10*. At the transcriptional level, fungal growth without copper overexpressed copper transporter of high affinity (*ctr1*) and the transcriptional factor, nitrogen metabolic repression (*nmr*), a signal of nitrogen-sufficient condition. Nitrogen-sufficient conditions could mainly regulate the *crt1* expression because, in nitrogen-limited conditions, laccase inducement by copper sulfate was lower than in carbon-limited conditions.

The transcriptome data also suggest that, in the GY4515 medium, the yeast extract and the concentration used could induce the metabolic repression of nitrogen. This result was corroborated by the overexpression of a transcript with an NMR-like domain. These results suggest repression by nitrogen could be related to the regulation of copper transporters that, once present in the culture medium, facilitate the entry of this metal into the fungal cell and induce the transcription of genes *lacc2*, *lacc6*, and *lacc10*. Furthermore, the *P. ostreatus* transcriptome from the GY4515 culture medium with copper sulfate revealed that the *lacc2*, *lacc6*, and *lacc10* genes responded to the addition of this laccase inducer under nutrient-sufficient conditions.

We explored the fungal microscopic-morphological changes in terminal hyphae, identified by the distal clamp connections, in culture media using ammonium sulfate or yeast extract. We observed interesting microscopic-morphological changes according to the sources of nitrogen and the C:N ratios. A large pattern of vacuoles was found using yeast extract. This behavior has also been observed in hyphae of *C. albicans* cultivated in nutrient-rich media, which showed fewer vacuolated compartments compared to the hyphae grown in low nitrogen media. Compared to that reported for this pleomorphic fungus, our results are similar [53]. The *P. ostreatus* hyphae grown in nutrient-rich media had fewer vacuolated compartments similar to those reported for *C. albicans*. Our study showed more vacuolated compartments grown in media with low nitrogen content in the culture media with higher C:N ratios. In another study, *P. ostreatus* was cultured in Potato-Dextrose-YE (PDY). The pellets from the culture media showed a young mycelium barely vacuolated (at the 5th culture day); however, when glucose concentration decreased for the culture time, the old mycelium was vacuolated (at the 12th culture day) [54]. Therefore, those vacuolated patterns in the young mycelium of *P. ostreatus* in SmF seem to signal nutrient-limitation in this fungus that could affect laccase activity.

Another sign of nutrient-sufficient conditions for carbon and nitrogen was tested, a culture medium whose composition increased biomass production using only glucose and YE in the culture medium. At the same time, the effect of glucose and YE concentrations on laccase activity in the absence and presence of 1 mM copper sulfate was also evaluated. The GY4515 medium was the composition closest to the maximum biomass production predicted by the statistical model. Surprisingly, the effect of the nutrient conditions in laccase activity was strongly dependent on copper sulfate in the culture medium.

Without copper sulfate, the glucose and YE concentrations were insufficient to explain the changes in laccase activity in the different culture media evaluated in this study. However, increasing the glucose concentration from 5 to 2 gL^−1^ resulted in a more than five-fold increase in laccase activity. An additional increase of up to 40 gL^−1^ in glucose concentration did not improve the laccase activity. On the contrary, lower activities were obtained [55]. In reports for another fungus, *Cerrena* sp., high carbon and nitrogen concentrations in the fermentation medium were beneficial for laccase production. In contrast, regardless of carbon concentration, low nitrogen concentrations drastically reduced laccase production [56]. However, the composition of the culture medium had little influence on laccase activity in *P. ostreatus* nutrient-sufficient conditions when the fungus grew in a culture medium with a lot of glucose 102.68 gL^−1^ and yeast extract 43.65 gL^−1^ concentrations, where the lignocellulosic biomass pretreatment and laccase activity improved the pretreatment system [57].

With copper sulfate, the changes in laccase activity in the different culture media were statistically explained by the glucose and YE concentrations evaluated using a CCD. Here we only tested a fixed concentration of copper sulfate (1 mM) because this was the concentration that had the highest increase in laccase activity when different copper sulfate concentrations (0.5 to 5 mM) were tested in *P. ostreatus* in SmF [58]. In other studies in *P. ostreatus* and other fungi, the design of experiments (DOE) of response surface methodology was also used; however, copper sulfate and nutritional factors were tested at various concentrations [52,55,59,60,61,62]. Despite this, the main finding was similar to the finding in this study: nutritional factors, mainly organic nitrogen source and copper concentrations, showed an interaction effect that increased the laccase activity considerably in the culture media. Therefore, evidence was collected to infer that fungal biomass in nutrient-sufficient conditions is more susceptible to laccases inducers than nutrient-limited culture conditions. However, when the profile of *P. ostreatus* growth in GY4515 was characterized with and without copper, the main difference was laccase activity. Furthermore, the local transcriptional analysis of the laccase gene family showed that *lacc2*, *lacc6*, and *lacc10* were overexpressed in the culture with copper.

The Zymogram results indicate that the isoenzymes induced by copper are affected by different nutrient conditions. In nitrogen-sufficient conditions that are also carbon-limited, the expression of isoenzymes around 70 KDa (Presumably lacc6) was negatively affected, while the isoenzymes Lacc2 and Lacc10 (around 40 KDa) were positively affected. All isoenzymes induced by copper were negatively affected by the inverse nutrient conditions (i.e., in carbon-sufficient conditions and nitrogen-limited). These results show an important effect of nitrogen-limited laccase activity induced by copper, suggesting that processes associated with organic nitrogen metabolisms influence copper uptake. In contrast, in nitrogen-sufficient that are simultaneously, the carbon-limited conditions, only some specific laccase isoenzymes induced by copper are affected. In these carbon-limited conditions, laccase activity is still influenced by copper because there is probably copper uptake.

Copper-induced laccase isoforms enzymes were previously identified in SmF [63]. The most abundantly secreted with the highest migration pattern Lacc10 (PoxC), then Lacc2 (PoxA3) showed an intermediate migration pattern [17], and Lacc6 (PoxA1b) was the one that presented the lowest migration pattern [40]. However, the zymogram analysis in this study showed four laccase isoenzymes. The transcriptome analysis found *lacc2*, *lacc6*, *lacc10*, *pox2*, and *PoxA3a* among the top 10 overexpressed transcripts. This last transcript does not code for a laccase enzyme but for a protein that can form heterodimers with Lacc2 (PoxA3) [17,64]. We do not know if any laccase heterodimeric was present in the analyzed enzymatic crude extract; however, four bands in the zymogram analysis suggest Lacc6, Lacc2, Lacc10, and another isoform.

It is noteworthy to observe how these isoforms changed in the nutrient-limited conditions in the culture medium. In carbon-limited conditions, the two bands at 70 KDa disappeared, while in nitrogen-limited conditions, two bands at 70 KDa and two bands at 40 KDa were drastically affected. These results may support the idea that nutrient-sufficient conditions confer the fungus more susceptibility to laccase inducers. A marker for nutrient-sufficient conditions was found on the *nmr1*-like transcript listed in the top ten transcripts in culture without copper. In these nutrient-sufficient conditions, three *ctr1* copper transporters were also found. This copper transporter has already been shown to negatively regulate CTR1 transcription in the dikaryon strain dkN001 and its monokaryon parents mkPC9 and mkPC15 of *P. ostreatus* [65].

Finally, using different nitrogen sources and C:N ratios allowed us to observe that nitrogen-limited conditions had a stronger relationship with the vacuolated patterns. Moreover, nitrogen-limited conditions affected laccase induction by copper. All these results again suggest a likely relationship between nitrogen metabolisms and copper uptake. In this relationship, *P. ostreatus* in initial nitrogen-limited conditions medium produce hyphal with vacuolated morphology. These hyphal could have a lower expression level of copper transporters such as ctr1 than hyphal obtained in the nitrogen- sufficient condition. Consequently, copper uptake mechanisms are less prevalent in nitrogen-limited than in nitrogen-sufficient conditions, affecting laccase genes regulation by transcriptional mechanisms in the cis-acting elements promoter sequences as metal responsive elements (MRE) [12].

## 5. Conclusions

This study describes a systematic analysis of the composition of the culture medium based on two nutritional factors: glucose as a carbon source and ammonium sulfate or yeast extract as nitrogen sources. This analysis determined what type of nitrogen source and what combination of glucose and yeast extract concentrations favored the production of fungal biomass and laccase activity induced by copper. The nutrient-sufficient conditions for both carbon and nitrogen in *P. ostreatus* cultures favored the expression of a transporter family of genes with a high affinity to copper: the CTRs, which were able to uptake copper from the environment. This effect was evidenced by an increase in copper-dependent laccase activity by 20-fold, the presence of four laccase isoforms that were detectable in zymogram and by an increase in three laccase transcripts *lacc2, lacc6,* and *lacc10* determined by real-time qPCR and RNAseq.

## Figures and Tables

**Table 1:** Forward and reverse primers sequence to for the laccase and reference genes [41].

Gene Name	Primer Sequence (Fw) ^1^	Primer Sequence (Rv) ^2^
*lacc1*	GGTACATCCTAGCACCCAATG	GACGAGATCAGTTTCCAAGAGG
*lacc2*	CCCTGGCAGATTGGTATCATG	ATGACAGCGTAAGGGACAAG
*lacc3*	TCGTTTCCGTCTCGTTTCTC	CTGCGAAGATTTGGATGCTGG
*lacc4*	CCCCATCCTTTCCATCTTCAC	TAGTTATACACCGAGCTTCCG
*lacc5*	CGCATTTGCCGCTTTCTT	GGTGACTAGGACTGAGTATCTC
*lacc6*	GTACAACTACGAAAACCCCG	CAAGGTCAAGATGCCAGT
*lacc7*	GTTGATAGCCTCCAGATCTTCG	GTAGGATGGCGGAGTTGATG
*lacc8*	CATTGGCTGTGACTCGAA	GGATCAGAGAATAGCGTTGG
*lacc9*	CTATCCTTCGGTATGCTGGTG	ATATTGATGTCTGCGCCTCC
*lacc10*	CCTACTTCCCCTTTGGCTATC	ATGACGAGCAAAGAGTGACC
*lacc11*	CCTGAATGGTCTGATCTCTGC	CCTATGACTTGGGCTCTTCG
*lacc12*	GTACTCATTTTCGGCTCCTG	CCACGTAGTCCATCGCAATA
*sar1*	GGATAGTCTTCCTCGTCGATAG	GGGTGCGTCAATCTTGTTAC
*gapdh1*	TGGTCCATCGCATAAGGA	ACACGGAAGGACAAACCA
*actin1*	AGTCGGTGCCTTGGTTAT	ATACCGACCATCACACCT
*Pep*	GATTCCAGAGGACAAGGACGCAA	AAATCTTCCGCGATACGGGTCACT
*lacc1*	GGTACATCCTAGCACCCAATG	GACGAGATCAGTTTCCAAGAGG

^1^ Fw: forward; ^2^ Rv: reverse.

**Table 2:** Biological growth parameters of *Pleurotus ostreatus* in culture media with ammonium sulfate or yeast extract in submerged fermentation.

Ammonium Sulfate (gL^−1^)	Yeast Extract (gL^−1^)	Glucose (gL^−1^)	C:N	Biomass (gL^−1^)	Final Glucose (gL^−1^)	Max. Laccase Activity (UL^−1^)
1.0	0	0.5	1:1	0.26 ± 0.02	0.18 ± 0.03	2.85 ± 2.31
1.0	0	1.0	2:1	0.39 ± 0.02	0.58 ± 0.02	3.40 ± 0.58
1.0	0	10.0	22:1	1.30 ± 0.18	6.86 ± 0.97	6.35 ± 3.07
10.0	0	10.0	2:1	1.18 ± 0.10	6.47 ± 0.51	1.74 ± 0.15
0	5.0	1.0	5:1	0.91 ± 0.04	0.06 ± 0.02	944 ± 71.6
0	5.0	10.0	14:1	3.62 ± 0.01	0.12 ± 0.04	489 ± 63.2
0	5.0	20.0	23:1	7.65 ± 0.08	1.16 ± 0.89	104 ± 10.1
0	10.0	20.0	14:1	7.04 ± 0.18	0.49 ± 0.13	2317 ± 787.7

**Table 3:** Biomass and maximum laccase activity in culture media of *P. ostreatus* with different concentrations of yeast extract and glucose from a central compound design.

Culture Name ^1^	Factors		Response Variables			
			Without Copper		With Copper Sulfate 1 mM	
	Glucose (gL^−1^)	YE (gL^−1^)	Biomass (gL^−1^)	Log_10_ MLA ^2^ (UL^−1^)	Biomass (gL^−1^)	Log_10_ MLA ^2^ (UL^−1^)
GY3008	30.0	7.50	9.25	2.69	10.4	3.82
GY6008	60.0	7.50	15.3	3.09	15.6	2.65
GY3023	30.0	22.5	11.2	2.76	9.71	4.22
GY6023	60.0	22.5	8.68	3.12	11.0	4.02
GY2415	23.8	15.0	12.2	3.18	10.9	4.17
GY6215	66.2	15.0	10.9	2.88	12.9	4.13
GY4504	45.0	4.39	5.29	1.99	6.13	2.22
GY4526	45.0	25.6	10.7	3.17	15.5	3.80
GY4515	45.0	15.0	16.6	3.11	22.4	4.50
GY4515	45.0	15.0	14.5	3.06	19.8	4.47
GY4515	45.0	15.0	16.9	3.12	19.4	4.48
GY4515	45.0	15.0	19.5	3.03	19.1	4.40
GY4515	45.0	15.0	16.5	2.99	19.0	4.41

^1^ MLA indicates the maximum laccase activity to the 21st day of culture. ^2^ G and Y stand for glucose and YE, respectively. The first two digits indicate the glucose concentration (gL^−1^), and the last two digits indicate the YE concentration.

**Table 4:** Transcript identification and annotations for the top 10 differential gene expression in *P. ostreatus* from cultures in GY4515 medium with or without copper sulfate.

JGI PC15 V2.0 Transcript Id.	JGI Annotations	Short Name	JGI PC15 V2.0 Transcript Id.	JGI Annotations	Short Name
GY4515 + Cu^2+^			GY4515		
1089723	Multicopper oxidase, laccase, Lacc10 (PoxC)	Lacc10	1095975 ^1^	Unannotated gene Similar to ctr1 gene for copper transporter, exons 1–3 *Pleurotus* sp. “Florida”	*ctr1*
1105204 ^1^	Unannotated gen. Similar to Phenol oxidase (*pox2*) gene	Pox2	159791	Unknown protein	Unk_1
1097654	Cupredoxin domin	Cup	1092022	Copper transporter, Ctr1p	Ctr1p
1113032	Multi-copper oxidase Laccase, Lacc6 (PoxA1b)	Lacc6	1086646	Unknown protein	Unk_2
1067572	Small subunit of laccase PoxA3a	PoxA3a	1088435 ^1^	Unannotated gene Similar to ctr1 gene for copper transporter, exons 1–3 *Pleurotus* sp. “Florida”	*ctr1*
1063469	Cytochrome P450 CYP2 subfamily	P450	1090041	Unknown protein	Unk_3
1087630	Blue (type 1) copper domain	Cu-d	1090781	Unknown protein	Unk_4
1105457	Predicted E3 ubiquitin ligase/Zinc finger, C3HC4 type	E3_Zn	1099858	Polyketide cyclase	PC
1067328	Multi-copper oxidases, Lacc2	Lacc2	171939	NmrA-like family	Nmr
1062660	Nuclear pore complex, Nup98 component (sc Nup145/Nup100/Nup116), and Cupredoxin domain	NPC98/Cup	1077411	Unknown protein	Unk_5

^1^ The Basic Local Alignment Search Tool (BLAST)’s, from The National Center for Biotechnology Information (NCBI), standards database sequences were used to find local similarity between JGI sequences unannotated.

## Data Availability

The data presented in this study are available upon request from the corresponding author. The data are not publicly available because other data from these whole-genome transcriptomes are being used for other analyses to be published independently of this one.
